# Correlation of Ischemia-Modified Albumin with SOFA and APACHE II Scores in Preoperative Patients with Colorectal Cancer

**DOI:** 10.1155/2014/959075

**Published:** 2014-12-08

**Authors:** Masaaki Satoh, Kazuhiko Kotani, Alejandro Gugliucci, Hisanaga Horie, Russell Caccavello, Mamoru Takeuchi

**Affiliations:** ^1^Department of Anesthesiology and Critical Care Medicine, Jichi Medical University, 3311-1 Yakushiji, Shimotsuke, Tochigi 3290498, Japan; ^2^Department of Clinical Laboratory Medicine, Jichi Medical University, 3311-1 Yakushiji, Shimotsuke, Tochigi 3290498, Japan; ^3^Glycation, Oxidation and Disease Laboratory, Touro University California, 1310 Club Dr, Vallejo, CA 94594, USA; ^4^Department of General Surgery, Jichi Medical University, 3311-1 Yakushiji, Shimotsuke, Tochigi 3290498, Japan

## Abstract

*Purpose*. Critical illnesses are assessed according to the sequential organ failure assessment (SOFA) and acute physiology and chronic health evaluation (APACHE) II. Circulating ischemia-modified albumin (IMA) is a biomarker generated under ischemic and oxidative conditions and may reflect disease severity in preoperative patients. This study investigated the correlations of IMA with SOFA and APACHE II scores in inpatients admitted for colorectal surgery. *Methods*. We examined 27 patients with advanced colorectal cancers (mean age 69 years, men/women = 15/12). Correlations between SOFA and APACHE II scores in addition to preoperative serum IMA and C-reactive protein (CRP) levels were analyzed. *Results*. The mean IMA level was 0.5 AU, and the median CRP level was 0.6 mg/dL. Median scores for SOFA and APACHE II were 2 and 12 points, respectively. Significant positive correlations between IMA and SOFA (*r* = 0.45, *P* < 0.05) and IMA and APACHE II (*r* = 0.45, *P* < 0.05) were identified which remained significant in confounder-adjusted analyses. In contrast, weak correlations were observed between CRP and the SOFA and APACHE II scores. *Conclusions*. The positive correlations between IMA and both SOFA and APACHE II scores suggest that serum IMA measurements reflect the severity of systemic failure in patients admitted for colorectal surgery in the preoperative phase.

## 1. Introduction

Albumin has a binding site of transitional metal ions, including cobalt and copper, on its N-terminal region [1]. Ischemic and oxidative stress modifies this terminal peptide irreversibly to a dysfunctional form, known as ischemia-modified albumin (IMA) [2, 3]. IMA has been studied as a surrogate of cardiac ischemia and as a marker related to critical pathologies, including cancers as well [4–9].

In critically ill patients, assessment based on disease severity is crucial for providing suitable treatments and improving outcomes; therefore, several scoring systems have been developed. The sequential organ failure assessment (SOFA) [10] and the acute physiology and chronic health evaluation (APACHE) II [11, 12] are available as scoring systems of disease conditions of patients undergoing surgical treatment and/or intensive care. The clinical implications of these scoring systems have been confirmed, though there are some problematic aspects (e.g., complexity of scoring), and the scoring systems are not perfect in patient assessment [13]. Thus, research on patient assessment using these scores should be further explored.

Colorectal cancer is common in modern societies, and the management of colorectal cancer is crucial [14]. Colorectal tumors have increased levels of different markers of oxidative stress, such as increased levels of reactive oxygen species and nitric oxide [15, 16]. A prior study reported that the levels of serum IMA and the number of diseased coronary arteries were positively correlated [17]. The increase of serum IMA levels was associated with the elongation of the seizure period [18]. Thus, the degree of oxidative stress is thought to be associated with disease severity. To date, the relationship between the IMA and the degree of severity in patients with colon cancers is unknown. Therefore, the current study investigated the correlation of IMA with SOFA and APACHE II scores in patients admitted for colorectal surgery.

## 2. Methods

A total of 27 patients were consecutively enrolled into the study. The study was conducted in accordance with the Declaration of Helsinki (1964) [19] and approved by the Ethics Committee of Jichi Medical University. The study was conducted at our university hospital in 2012. Included were patients for whom surgery was indicated for advanced colorectal cancer as well as colonic obstruction and peritonitis due to cancer. Informed consent was obtained in the presurgical phase.

For each patient, esophageal temperature was recorded. Blood pressure and heart rate were determined using an automated sphygmomanometer (BSM-6701; Nihon Kohden Corp., Tokyo, Japan). The respiratory rate and the Glasgow coma scale score [20] were evaluated before induction of anesthesia. Clinical conditions, such as the use of inotropes and underlying diseases (i.e., chronic organ insufficiency, immunodeficiency, diabetes mellitus, and ischemic heart disease), were based on information obtained from the patients' medical charts.

Arterial blood gas analysis (electrode method) was performed to determine pH, arterial oxygen partial pressure (PaO_2_), and alveolar-arterial oxygen difference (A-aDO_2_). Hematocrit, leukocyte counts, and platelet counts were determined by an autoanalyzer (Beckman Coulter, LH800 system, Tokyo, Japan). Plasma sodium and potassium levels were determined with a selective electrode method. Serum bilirubin levels were determined with an enzymatic method. These were all measured at our single, central hospital laboratory. Blood samples were measured at 3 days before operations in patients with the elective surgery, whereas all measurements were performed at least 1 hour before the emergency operation started.

Serum CRP levels were determined with an enzyme-linked immunosorbent assay (Eiken Chemical Co. Ltd., Tokyo, Japan). Serum IMA levels were determined with cobalt^2+^-binding reduction in our laboratory, as published previously [4]. Briefly, a 100 *μ*L serum sample was added to a 25 *μ*L solution of 1 g/L cobalt chloride. Then, dithiothreitol (25 *μ*L of a 1.5 g/L solution) was added. The absorbance of the mixture was recorded at 470 nm, and the IMA level was expressed as the delta absorbance in arbitrary units between the individual control (without dithiothreitol) and the reaction sample.

The SOFA score was developed to evaluate the degree of organ dysfunction [10]. The SOFA scoring scheme assigns 1 to 4 points to each of the levels of dysfunction of six organs (respiratory, circulatory, renal, hematological, hepatic, and central nervous systems). The following components are included: PaO_2_/FiO_2_, mean blood pressure, serum creatinine or daily total urine volume, platelet count, serum bilirubin, and Glasgow coma scale score. A score of 0 to 24 points is assigned, and a higher score is associated with higher severity [10].

The APACHE II score is evaluated based on the initial score of 12 components, with a distribution of 0 to 71 points. The severity is higher when the score is higher [11]. The evaluation is composed of three parts, including physiologic scores ranging from 0 to 60 points, age scores ranging from 0 to 6 points, and comorbid disease scores ranging from 0 to 5 points. The physiologic parameters include body temperature, mean blood pressure, heart rate, respiratory rate, PaO_2_, A-aDO_2_, pH, serum sodium, serum potassium, serum creatinine, hematocrit, white blood cell count, and Glasgow coma scale score. In general, the APACHE II score is used in patients treated in the intensive care unit (ICU). However, a previous report has shown the usefulness of preoperative application of the score to evaluate postoperative morbidity and death among patients undergoing elective surgery [21]. Evaluating a single SOFA score alone may be of dubious value; therefore, in the current study, the authors decided to use the preoperative APACHE II score in addition to the SOFA score, in order to guarantee the disease severity of the studied patients.

Data are expressed as means ± standard deviation or medians with interquartile ranges. The correlations between the IMA or CRP levels and the other variables were examined using Pearson's correlation test. When the correlation test was significant, a subsequent multiple regression analysis, adjusted for confounders such as age and sex (Model 1) as well as age, sex, and the presence of diabetes mellitus and ischemic heart disease (Model 2), was also used to examine the correlations between the IMA and CRP levels and the other variables. All variables with skewed distributions were log-transformed for all analyses. The data were analyzed using SPSS 17.0 software (SPSS Inc., Chicago, IL, USA). A *P* value < 0.05 was considered significant.

## 3. Results

**Figure 1:**
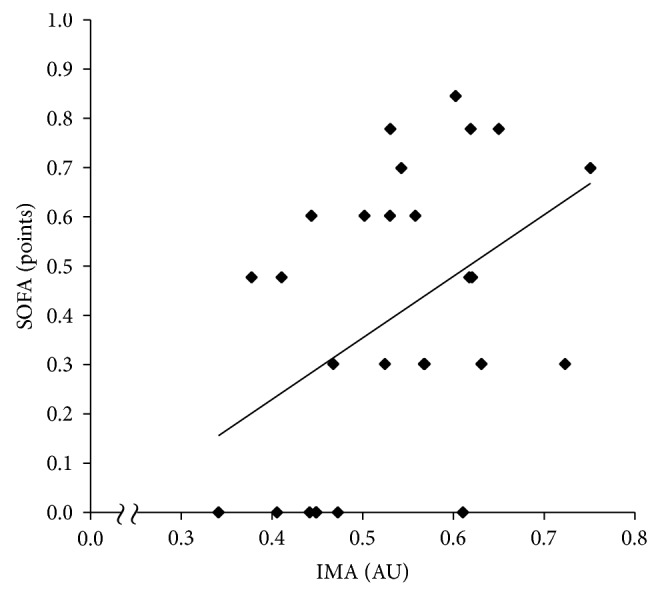
Correlation between IMA and SOFA. IMA: ischemia-modified albumin, SOFA: sequential organ failure assessment. The SOFA score is log-transformed.

**Figure 2:**
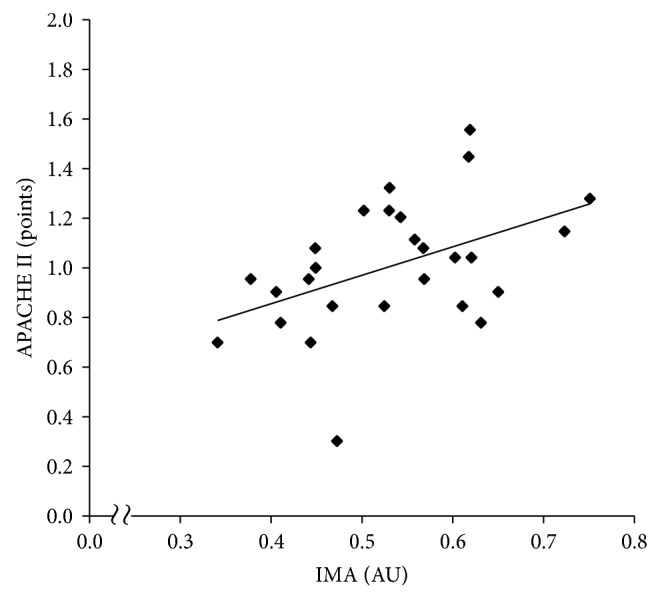
Correlation between IMA and APACHE II. IMA: ischemia-modified albumin, APACHE: acute physiology and chronic health evaluation. The APACHE II score is log-transformed.

The clinical data are shown in Table 1. The study population included eight patients with diabetes mellitus and one patient with a previous history of ischemic heart disease. There was a significant positive correlation between the SOFA and APACHE II scores (*r* = 0.54, *P* < 0.01). As shown in Table 2 and Figures 1 and 2, there were significant positive correlations between IMA and SOFA (*r* = 0.45, *P* = 0.02) and between IMA and APACHE II (*r* = 0.45, *P* = 0.02). The positive correlation between IMA and SOFA remained significant (Model 1: *β* = 0.48, *P* = 0.02; Model 2: *β* = 0.50, *P* = 0.02) in the confounder-adjusted analyses. The positive correlation between IMA and APACHE II also remained significant (Model 1: *β* = 0.44, *P* = 0.02; Model 2: *β* = 0.53, *P* = 0.02) in the confounder-adjusted analyses.

A significant positive correlation between IMA and CRP levels (*r* = 0.41, *P* = 0.04) was observed, and their positive correlation also remained significant (Model 1: *β* = 0.41, *P* = 0.04; Model 2: *β* = 0.43, *P* = 0.04) in the confounder-adjusted analyses. However, weak correlations were observed between CRP and the SOFA and APACHE II scores, as shown in Table 2.

## 4. Discussion

The current study demonstrated that the IMA was significantly and positively correlated with SOFA and APACHE II sores in preoperative patients awaiting colorectal surgery. The findings are the first to indicate that serum IMA measurements can reflect the severity of systemic failure, as evaluated in the scoring systems, among patients with colorectal cancers. Since these scores are established and commonly used in clinical settings [10, 11, 20], the findings regarding the IMA appear to be reliable, and the preoperative IMA levels may help clinicians assess these patients.

Patients with colorectal cancers have pathological abnormalities related to oxidative stress [22]. In fact, patients with several types of cancers can have high IMA levels [23–25]. Hypoxia/ischemia induces IMA by cleavage of the first two amino acids (Asp-Ala) of the HSA N-terminus, which is recognized as a strong binding site for transitional metal ions. [26, 27]. Patients with systemic complications (i.e., bacteremia, hepatic, or renal failure) would have enhanced oxidative stress [3]. These processes offer a possible mechanistic explanation for the correlation between the IMA and severity scores.

In analyzing the respective components of the SOFA or APACHE II scores, there was a significant correlation between the IMA and high heart rate or existing hypotension (data not shown). These component parameters are possibly involved in the degree of oxidative stress; accordingly, these could be particularly important determinants of increased IMA levels.

In regard to the use of the SOFA and APACHE II scores, there is some concern that these scoring systems are complex [13]. On the other hand, the IMA is easily and inexpensively measurable. Moreover, these scores are not perfect in predicting postoperative outcomes [13]. IMA levels have been reported to be predictive of patients' outcomes (in particular, short-term outcomes) [28–31], though most studies examined cardiovascular diseases as outcomes given the earlier knowledge about IMA [32]. The use of IMA, plus the SOFA and APACHE scores, for the assessment of critically ill patients in association with outcomes, may be a future issue.

The CRP is a well-known inflammatory marker to assess patients with critical illnesses [33]. The significance of CRP measurement in preoperative patient assessment is recognized to be limited, while, for instance, there was a report showing the usefulness of CRP in patients with bacterial infections [33]. Because the CRP is synthesized in the hepatic cells within 6-to-8 hours after infection [34] and thereafter the peak of CRP concentrations exists in the circulation between 36 and 50 hours [35], there may be a gap between increased serum CRP levels and infectious states. The production of IMA is induced by the direct stimuli of oxidative stress, and the serum IMA levels can be increased immediately within an hour [26]. Thus, the current study results showing weaker correlations of CRP with SOFA and APACHE II scores, compared to IMA, are not unexpected. In comparison with oxidative stress marker IMA, CRP could not reflect the degree of critical illness severity at the point.

The current study has some limitations. The sample size was small. The patients were only those awaiting colorectal surgery and values of APACHE II and SOFA scores revealed that most of the patients did not have a severe systematic failure/dysfunction; therefore, careful attention must be paid when attempting to generalize the current results. The coexisting coronary artery disease and ischemic heart disease had not completely been evaluated in this study. Although the APACHE II score is generally used in patients treated in the ICU, its use was extended to the preoperative phase in the current study. We acknowledge that there may be some differences relative to the more general use of the score. In this sense, the current work preliminarily offers a proof of principle of the validity of IMA in these cases and should open the way for future studies.

## 5. Conclusions

In summary, the study results that showed positive correlations between IMA levels and both SOFA and APACHE II scores suggest that serum IMA can reflect the severity of systemic failure among patients with colorectal cancers in the preoperative phase. The easy practicality of the assay will facilitate future studies looking at the predictive value of IMA in such patients.

## Figures and Tables

**Table 1:** Clinical data of the studied patients.

Variable	Levels
Age, years	69 ± 12
Men : women	15 : 12
IMA, AU	0.53 ± 0.10
CRP, mg/dL	0.6 (0.1–5.5)
SOFA, points	2 (0–3)
APACHE II, points	12 (7–16)
Body temperature, °C	36.6 ± 0.7
Mean blood pressure, mmHg	90 ± 23
Hypotension or use of an inotrope, *n*	6 (22%)
Heart rate, bpm	86 ± 24
Respiratory rate, breaths/min	20 ± 8
A-aDO_2_ (FiO_2_ > 50%) or PaO_2_ (FiO_2_ < 50%), mmHg	375 ± 121
pH	7.4 ± 0.9
Plasma sodium, mmol/L	139 ± 4
Plasma potassium, mmol/L	4.3 ± 0.8
Serum creatinine, mol/L	80 (53–114)
Acute renal failure, *n*	2 (0.7%)
Hematocrit, %	34.8 ± 6.5
Leukocytes, ×10^9^/L	6.7 (4.7–12.3)
Platelets, ×10^9^/L	239 ± 86
Bilirubin, mol/L	12 (9–14)
Glasgow coma scale, points	14.4 ± 1.6
Organ insufficiency or immunodeficiency, *n*	13 (48%)

IMA: ischemia-modified albumin; CRP: C-reactive protein; SOFA: sequential organ failure assessment; APACHE: acute physiology and chronic health evaluation.

Data are expressed as means ± standard deviations, medians (interquartile ranges), or patient numbers.

**Table 2:** Correlation coefficients of IMA or CRP with other variables related to APACHE II or SOFA.

Variables	IMA	CRP
Age, years	0.05 (0.79)	0.18 (0.37)
Men	−0.03 (0.90)	0.01 (0.98)
SOFA, points	0.45 (0.02^*^)	0.26 (0.19)
APACHE II, points	0.45 (0.02^*^)	0.33 (0.09)

IMA: ischemia-modified albumin; CRP: C-reactive protein; SOFA: sequential organ failure assessment; APACHE: acute physiology and chronic health evaluation. Data are expressed as simple correlation coefficients (*P* values). ^*^ *P* > 0.05.
